# Considerations regarding a prediction model of surgical site infection after gastrointestinal surgery

**DOI:** 10.1097/JS9.0000000000001426

**Published:** 2024-04-09

**Authors:** Shenglin Zhao, Hong Zhou, Mei He

**Affiliations:** a Office of Chongqing Cancer Prevention and Treatment; b Chongqing Key Laboratory of Translational Research for Cancer Metastasis and Individualized Treatment, Chongqing University Cancer Hospital, Chongqing, People’s Republic of China

We read with great interest the article ‘Prediction models of surgical site infection after gastrointestinal surgery: a nationwide prospective cohort study’ by Yang *et al*.^1^, recently published in the *International Journal of Surgery*. The authors used multicenter data from 57 hospitals and 17 353 participants to construct a prediction model for postoperative surgical site infection (SSI) after gastrointestinal surgery. The model included 20 variables, including demographics, preoperative blood biochemical parameters, comorbidities, immunosuppressive agents, preoperative preparation, and surgical information, to distinguish the risk of SSI in patients. This multicenter, representative predictive model provides a feasible method for preventing SSI occurrence in China. However, we have discussed a few issues related to this study.

First, regarding the development of the predictive model, the authors seemed to have overlooked the impact of data leakage. The authors performed imputation and univariate analysis on the entire dataset before splitting it into training and testing sets. The leakage of information from the test set into the feature selection process introduces spurious correlations between the training and test sets, leading to overly optimistic estimates of model performance^2^. A more rigorous approach would be to perform data imputation, univariate analysis, and feature selection on the training set after data splitting and then evaluate the model’s performance on the test set using the selected features.

Second, the study included data from 57 hospitals to ensure representativeness of the sample. However, the authors did not address the potential heterogeneity among centers or describe how they handled this issue during data splitting. There were significant differences in SSI occurrence levels among hospitals with different grades. If samples from the same center are present in both the training and test sets, the model may learn center-specific features, resulting in overly optimistic performance on the test set^3^. The authors should clarify whether they have employed strategies such as stratified splitting or center-level analysis to mitigate this concern.

Finally, although the model was developed to predict the risk of SSI after gastrointestinal surgery, the results showed that colon surgery had an odds ratio (OR) >1, while appendix surgery had an OR <1. This finding suggests that there may be differences in SSI risk between colon and appendix surgeries, warranting further investigation^4,5^. This discrepancy could be due to differences in preoperative preparation, surgical techniques, patient characteristics, or postoperative management between the two types of surgeries. Additionally, interaction effects or unmeasured confounding factors may contribute to the observed differences in SSI risk. To address this issue, we recommend conducting stratified analyses by surgery type, exploring the interaction effects, performing sensitivity analyses, and validating the model on external datasets. These approaches can help clarify the differences in SSI risk between gastric and intestinal surgeries and improve the predictive performance of the model.

Overall, we appreciate the contribution of the authors to research on SSI prevention. Their multicenter study made significant progress in this field of clinical relevance. However, we respectfully raise our concerns, which may help improve the clarity of their research findings.

## Ethical approval

Not applicable.

## Consent

Not applicable.

## Sources of funding

This study was funded by the First batch of key Disciplines on Public Health in Chongqing, China, the Early Gastrointestinal Cancer Physicians Joint Growth Program (GTCZ-2023-CQ-02), and the Medical Scientific Research Project of Shapingba District of Chongqing (Joint project of Chongqing Shapingba District Health Commission and Science and Technology Bureau, 2023SQKWLH016).

## Author contribution

S.Z.: writing the paper; M.H. and H.Z.: study design. All authors approved the final version of the manuscript.

## Conflicts of interest disclosure

There are no conflicts of interest.

## Research registration unique identifying number (UIN)

Not applicable.

## Guarantor

Mei He.

## Data availability statement

Not applicable.

## Provenance and peer review

Not commissioned, externally peer-reviewed.

## Presentation

Not applicable.

## Assistance with the study

Not applicable.
